# Survival prognosis and variable selection: A case study for metastatic castrate resistant prostate cancer patients

**DOI:** 10.12688/f1000research.8427.1

**Published:** 2016-11-16

**Authors:** Søren Wengel Mogensen, Anne H. Petersen, Ann-Sophie Buchardt, Niels Richard Hansen

**Affiliations:** 1 Department of Mathematical Sciences, University of Copenhagen, Copenhagen, Denmark

**Keywords:** imputation, lasso, generalized additive models, stability selection, survival forests, survival prognostic models

## Abstract

Survival prognosis is challenging, and accurate prediction of individual survival times is often very difficult. Better statistical methodology and more data can help improve the prognostic models, but it is important that methods and data usages are evaluated properly. The Prostate Cancer DREAM Challenge offered a framework for training and blinded validation of prognostic models using a large and rich dataset on patients diagnosed with metastatic castrate resistant prostate cancer. Using the Prostate Cancer DREAM Challenge data we investigated and compared an array of methods combining imputation techniques of missing values for prognostic variables with tree-based and lasso-based variable selection and model fitting methods. The benchmark metric used was integrated AUC (iAUC), and all methods were benchmarked using cross-validation on the training data as well as via the blinded validation. We found that survival forests without prior variable selection achieved the best overall performance (cv-iAUC = 0.70, validation-iACU = 0.78), while a generalized additive model was best among those methods that used explicit prior variable selection (cv-iAUC = 0.69, validation-iACU = 0.76). Our findings largely concurred with previous results in terms of the choice of important prognostic variables, though we did not find the level of prostate specific antigen to have prognostic value given the other variables included in the data.

## Introduction

The Prostate Cancer DREAM Challenge ^1, 2^, launched March 16, 2015, was a prediction competition, which challenged the participating teams to develop better prognostic models for survival of patients with metastatic castrate resistant prostate cancer. The full competition was divided into two subchallenges, with subchallenge 1a and 1b on prediction of survival and subchallenge 2 on prediction of discontinuation of treatment. Three of the authors of this paper participated in the challenge (as part of team KUStat) and made a final submission for subchallenge 1. We report here our findings and methodology developed for subchallenge 1a as well as in subsequent work carried out after the final submission.

The Prostate Cancer DREAM Challenge offered a large and complex dataset from four clinical trials containing data for around 2000 patients and with more than 100 potential predictor variables. The participating teams were free to develop any model, but predictions – in terms of *risk scores* – were assessed and compared in a fixed framework via submissions of predictions through a web interface. For the assessment data used, the survival status was held back from the participants, but the assessment system was fully disclosed, and we as participants could mimic the assessment procedure on the released data to optimize predictive performance.

A reference prognostic model existed ^3^ when the challenge was launched, and it was a requirement that the top-performing team could demonstrate an improvement over this reference model. The main scoring metric for assessing the prognostic models was time integrated AUC (iAUC). Halabi and coauthors ^3^ reported iAUC estimates of 0.73 and 0.76 for the reference model on a test and validation dataset, respectively.

The Prostate Cancer DREAM Challenge included three rounds of submissions to a leaderboard prior to the final submission, and we submitted predictions for the second round of the leaderboard, which achieved an iAUC of 0.8062. This appeared to be a clear improvement of the reference model, which achieved an iAUC of 0.7782 in the same leaderboard round. Our final submission achieved an iAUC of 0.7732, which placed our team roughly in the middle of a big group of 15 teams that achieved an iAUC between 0.77 and 0.78 and well above the reference model, which achieved an iAUC of 0.7429 in the final scoring. However, the winning team managed to distinguish itself from the rest with an iAUC of 0.7915.

Our submission was based on a variable selection method called stability selection and a subsequent fit of a generalized additive model. Some ad hoc modifications were made, but it was unclear if they had any positive effect on the predictive strength of the model. We also experimented with different techniques for imputation as there are a large number of missing values in the dataset for some variables. The effect of the imputation technique was, however, not fully understood, though we suspected that more sophisticated imputation techniques had a negative effect on predictive performance.

In this paper, we report a systematic evaluation of a total of 24 combinations of methods for model fitting, variable selection and imputation. These include the methods we used for our participation in the Prostate Cancer DREAM Challenge, some methods that we tried, but found inferior, and some additional methods that we afterwards found could potentially improve on the generalized additive model. The paper is organized as follows: first we present some descriptive and exploratory aspects of the dataset, and we describe how the dataset was prepared for the model building and evaluation process; then we briefly describe all the different methods we considered, the R functions and packages that implement the methods used and the analysis pipeline; finally, we present our results and conclusions.

## Data

The Prostate Cancer DREAM Challenge dataset comprises patient baseline data as well as extensive longitudinal data tables from the comparator arm of four clinical trials: ASCENT-2 ^4^, MAINSAIL ^5^, VENICE ^6^, and ENTHUSE-33 ^7^. We will in this paper only consider the use of baseline variables for survival prognosis. Data from three of the four trials was released as *training data* for the Prostate Cancer DREAM Challenge, see Table 1, which includes followup survival and treatment discontinuation information. Data from the fourth trial (ENTHUSE-33, 470 patients) was released for leaderboard (157 patients) and final scoring (313 patients), and did not include followup survival information. The latter dataset comprising the 313 patients from the ENTHUSE-33 trial will be referred to as the *validation data*. Though we have not had access to survival times for the validation data, predictions for the validation data could be assessed via the Prostate Cancer DREAM Challenge web interface.

**Table 1.** Number of patients and registered deaths from the three clinical trials in the training data.

Trial	Nr. of patients	Nr. of deaths
ASCENT-2	476	138
MAINSAIL	526	92
VENICE	598	433
Total	1600	663

**Figure 1.**
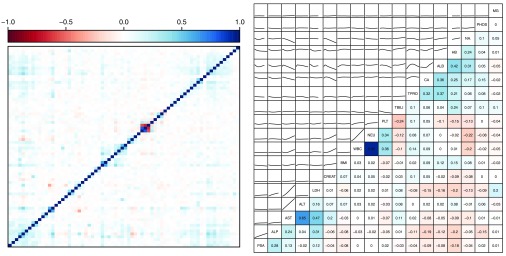
Correlation plot (left) for all binary predictors. See Supplementary Figure 1 for the correlation plot with labels. Correlations (right, below the diagonal) and pairwise associations as given by loess scatter plot smoothers (right, above the diagonal) for the numerical predictors.

We note that the survival distributions for the three trials in the training data are comparable, see Figure 1 in 2 (the *p*-value for the log-rank test of equal survival functions is 0.63), but we also note that the followup time for the VENICE trial was considerably longer than for the other two trials.

To assess prognostic models, it is important to understand the target population. The patients included in the four trials were not necessarily recruited from the same population, and Table 2, Table 3, and Table 4 present breakdowns of the patients in the four trials according to age group, geography, and race, respectively. We note that the age distribution for the ASCENT-2 trial differs from the other three trials with a larger proportion of patients over 75 years old and a smaller proportion in the group 18–64 years. The age distributions for the other three trials are similar. We also note that the majority of patients are white and from Europe or North America. For the ASCENT-2 trial there is no geographic data, but it is known that these patients were recruited from North America and Europe ^4^. The ASCENT-2 trial is, furthermore, the only trial for which the ethnicity group "Hispanic" is registered as race. A notable difference between the trials is that the validation data from the ENTHUSE-33 trial contains a larger proportion of Asians, and there are apparently no patients from the Asian/Pacific region in the training data. A further breakdown of the geographic group "Other" shows that the majority of these patients are white, but 30 patients from the VENICE trial are Asians; therefore they could be from the Asian/Pacific region. Nevertheless, there is little variability in the data in terms of race and geographic region.

**Table 2.** Number of patients stratified according to age group and trial.

	ASC.-2	MAINSAIL	VENICE	ENT.-33	Total
18–64	111	171	219	111	612
65–74	211	246	254	141	852
≥75	154	109	125	61	449
Total	476	526	598	313	1913

**Table 3.** Number of patients stratified according to geographic region and trial.

	ASC.-2	MAINSAIL	VENICE	ENT.-33	Total
W. Europe	0	247	212	104	563
N. America	0	139	80	61	280
E. Europe	0	84	127	50	261
S. America	0	0	86	38	124
Asia/Pacific	0	0	0	47	47
Africa	0	0	0	13	13
Other	0	50	93	0	143
Missing	476	6	0	0	482
Total	476	526	598	313	1913

**Table 4.** Number of patients stratified according to race and trial.

	ASC.-2	MAINSAIL	VENICE	ENT.-33	Total
White	419	433	538	225	1615
Asian	5	0	36	49	90
Black	32	25	17	12	86
Hispanic	14	0	0	0	14
Other	6	13	7	27	53
Missing	0	55	0	0	55
Total	476	526	598	313	1913

All the baseline values for the predictor variables were made available for the Prostate Cancer DREAM Challenge participants in a data table referred to as CoreTable. The variables in CoreTable were filtered and cleaned prior to the model building. The filtering consisted of excluding variables that were mostly or entirely missing in either the training or the validation data, or had no variation. Variables related to treatment and discontinuation were excluded as well. The cleaning consisted of consistent registration of missing values, correct registration of variable type (categorical or numeric), and some recoding. The filtered and cleaned data contains 93 predictor variables in addition to the followup survival time, the death indicator, and the patient ID, see Table S1. Of the 93 predictors 72 are binary predictors, 4 are categorical predictors with three or more categories, and 20 are numerical predictors, which, except for BMI, are laboratory measurements. Table S1 shows that there are a considerable number of missing values in the training data for some of the numerical variables, while the validation data set is more complete. In fact, 37 of the 63 missing values in the validation data are related to only two patients, for whom most laboratory measurements are missing.

**Supplementary Figure 1.**
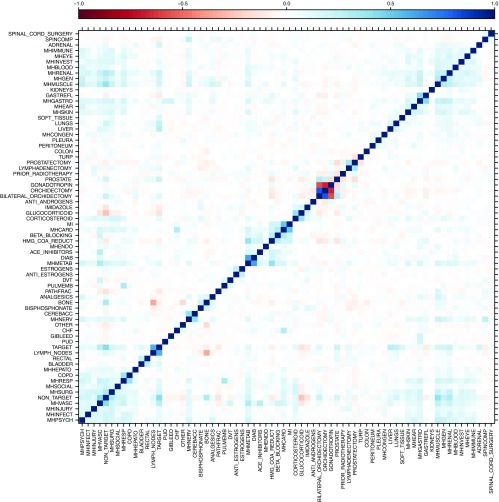
Correlation plot for all binary predictors.

Figure 1 shows pairwise correlations between binary predictors and correlations and associations between the numerical predictors. The variables were ordered by hierarchical clustering based on the correlations. We note that there is some correlation among the predictors. Among the numerical predictors, the variables CA, ALB, HB and NA. are positively correlated and also correlated with total protein TPRO. The variables NEU (neutrophils, a white blood cell type) and WBC (white blood cells) are unsurprisingly strongly positively correlated, and they are both positively correlated with PLT (platelets). Finally, the group PSA, ALP, AST, ALT and LDH also shows positive correlations with AST (aspartate aminotransferase) and ALT (alanine transaminase) being strongly correlated. For the binary variables, it is worth noting that the correlation pattern is rather weak and does not cluster in any clear pattern, though there is a certain weak overall positive correlation pattern. We see this pattern most clearly for the variables related to medical history (see Supplementary Figure 1 at the end of the paper for labels with medical history variables having prefix MH). Some of the strongest clustered correlations are unsurprising, such as the positive correlation among MI, MHCARD and BETA_BLOCKING related to cardiac disorders, the positive correlation between the metabolism and diabetes variables MHMETAB and DIAB, and the negative correlation between GONADOTROPIN (fertility medication) and ORCHIDECTOMY (testicle(s) removed).

## Methods

### Imputation

As mentioned above, the training data contains a large number of missing values. To deal with the missing values we implemented three imputation schemes: imputation under the missing completely at random assumption (MCAR), imputation under the missing at random assumption using only other predictors (MAR), and imputation under the missing at random assumption using other predictors as well as the survival response (MARwR). The variable to be imputed is denoted VI in the following.

The MCAR assumption means, as the name suggests, that the mechanism resulting in missing values is completely random and independent of both observed and unobserved variables. The corresponding imputation scheme is implemented by drawing observations randomly from the empirical marginal distribution of VI.

The two other imputation schemes involve fitting regression models with VI as the response variable, and their implementations share a number of components. Both schemes can use up to five other variables from the dataset to fit a linear regression model of VI. The five predictors are selected as the variables having the strongest marginal association with VI measured in terms of *p*-values. Only variables with *p* ≤ 0.05 and no missing values are considered. Missing values of VI are then imputed from the fitted regression model. The MAR scheme uses only predictor variables whereas the MARwR uses the censored survival times as well. Our MARwR scheme follows the suggestions by White and Royston ^8^ to use the Nelson-Aalen estimate of the cumulative hazard function together with the indicator variable for censoring in the imputation model of VI.

We did not implement a specific MAR or MARwR scheme for categorical variables, and the missing values of RACE_C and REGION_C were therefore imputed using the MCAR scheme.

### Proportional hazards models

All methods considered except random survival forests are based on the proportional hazards model with the hazard function for the *i*th patient being

$$\lambda_{i}(t)=e^{f(x_{i})}\lambda_{0}(t).$$

Here *λ*_0_ is a baseline hazard function and *f* is a function of the vector of predictor variables, **x**_*i*_ = ( *x _ij_*) *_j_* , for the *i*th patient. We refer to *f* ( **x**_*i*_) as the *risk score* for the *i*th patient. For the purpose of risk prediction in the context of the Prostate Cancer DREAM Challenge, any monotonically increasing transformation of *f* ( **x**_*i*_) – e.g. exp( *f* ( **x**_*i*_)) – is an equivalent risk score.

The additive, linear model is given by

$$f(x_{i})=\sum_{j=1}^{p}\beta_{j}x_{ij}.$$

The coefficients *β _j_* can be estimated by maximizing Cox’s partial likelihood using the function coxph from the survival R package ^9, 10^. However, for a large number of predictors there will usually be a favorable bias-variance tradeoff by using shrinkage and/or variable selection. Moreover, the additive, linear model may not be adequate, since it does not capture nonlinear or interaction effects on the log-hazard scale.

The generalized additive model is given by

$$f(x_{i})=\sum_{j=1}^{p}f_{j}(x_{ij}),$$

for functions *f _j_* of the univariate predictors. For numerical predictors the functions *f _j_* are generally assumed to be smooth. The model can be fitted to data by minimizing the negative log-partial-likelihood with a quadratic penalty that penalizes roughness of the *f _j_*-functions. This can be achieved by the function gam with family = cox.ph() from the mgcv R package ^11^. The function gam automatically chooses the trade-off between likelihood and penalty (and hence the smoothness) via built-in optimization of an unbiased risk estimate.

### Lasso

Lasso is a shrinkage and selection estimator that fits a proportional hazards model by minimizing the negative log-partial-likelihood with an *ℓ*_1_-penalty. The lasso estimator can be computed using the function glmnet with family = "cox" from the glmnet R package ^12, 13^. It fits models for a sequence of penalty parameters (the lasso path), and it supports selection of the penalty parameter via built-in cross-validation. Any choice of the penalty parameter will generally lead to some coefficients shrunk to 0, which can be interpreted as a variable selection procedure. For all the results presented in this paper, the penalty parameter for lasso was chosen by minimizing the cross-validated partial likelihood loss.

Lasso, with the penalty chosen as describe above, yields an additive, linear model and gives resulting estimates of the risk score. Some coefficients are shrunk to 0, hence lasso does implicit variable selection, but the coefficients for the selected variables are, in addition, shrunk toward 0. The debiased lasso re-estimates the coefficients for the lasso selected variables without shrinkage, and can be computed by coxph based on the variables selected by lasso.

### Stability selection

Stability selection ^14^ is a variable selection method that works by choosing variables that are stably selected on subsampled data by e.g. lasso. The method implemented is a slight adaptation of the method proposed by Meinshausen and Bühlmann in 14, which works as follows: The lasso path is computed for a subsample of the training data, cross-validation is used on the subsample to select the optimal penalty, and the coefficients not shrunk to 0 are selected for the subsample. To obtain the results reported in this paper we used the procedure with 100 replications and with each subsample being half the size of the full training data. The selection frequency was computed for all variables, and a cutoff for stably selected variables was chosen to be 50%.

Any method for fitting a survival regression model can be combined with stability selection by fitting the model using only the stably selected variables.

### Stochastic gradient boosting

A gradient boosting machine fits *base learners* sequentially to so-called pseudo-residuals. A base learner is a simple model of *f*, e.g. one small regression tree, and the ensemble estimate of *f* consists of an aggregate of all the base learners. Regularization by shrinkage may be applied for each base learner. A *stochastic* gradient boosting machine samples (without replacement) for each iteration a subset of observations uniformly from the training data and uses only this subset for fitting a particular base learner ^15^.

We used an implementation of a stochastic gradient boosting machine with trees as base learners that directly optimizes a smoothed version of the concordance index (C-index) as described in 16. The implementation is available on GitHub ^17^, which is a fork of an earlier version of the gbm R package ^18^. This implementation implicitly applies shrinkage when fitting an individual tree, as an optimal solution is not guaranteed ^16^. Pilot experiments indicated that additional explicit shrinkage did not improve the predictions, and therefore our implementation does not use explicit shrinkage. The subsampling fraction (bag fraction) controls the number of observations used for each tree fit. Our implementation sets the subsampling fraction to 0.5, allows for interactions of up to three variables, and uses a minimum node size of 10. The number of trees is chosen by built-in cross-validation with a maximum of 1000.

We fitted gradient boosting machines using all 93 predictor variables in the dataset as well as using only the stably selected variables.

### Random survival forests

A random survival forest is an ensemble method similar to a boosting machine that uses trees as base learners ^19^. For each iteration of the algorithm, a dataset of the same size as the original is sampled with replacement. A tree is then grown using this data set. For each node of the tree, a subset of variables is sampled and considered for splitting. The splitting is done according to one of the variables in order to maximize survival difference as measured by the log-rank test statistic. In each terminal node, a Nelson-Aalen estimate is calculated and the estimates are then aggregated into an ensemble fit of the cumulative hazard function. To obtain a single predicted outcome for each subject, we used *ensemble mortality* as defined in 19.

Random survival forests can be fitted using the randomForestSRC R package ^19^. Our implementation uses 1000 trees with a minimum node size of 6 (number of events in terminal nodes). For each split the procedure considers 20 candidate variables, and for each of those variables a maximum of 10 potential splitting points are randomly chosen. Setting a maximum of potential splitting points has two purposes. First, it speeds up computations. Second, it counters the fact that the algorithm is biased towards splitting on continuous variables as opposed to variables with only a few levels ^20^.

As for stochastic gradient boosting, survival forests were fitted using all predictor variables as well as only the stably selected variables.

### The score metric

Survival prognosis can be viewed as a prediction of a binary variable (is the patient dead) at each future time point, in which case the prognosis by the risk score can be evaluated using the conventional AUC score at any given time point. The time integrated AUC (iAUC) constitutes a single summary score, and it was the main score metric for the DREAM subchallenge 1a. The score can be estimated using the timeROC R package ^21^.

### The modeling and assessment pipeline

The methods described above for imputation, for fitting a survival model, and for variable selection can be combined in a number of ways. We implemented all meaningful combinations resulting in a total of 24 prognostic models, see Table 5.

**Table 5.** The eight used combinations of variable selection and methods for fitting a survival model. All eight combinations were used in combination with all the three imputation methods: MCAR, MAR and MARwR.

Method	All variables	Stab. selected variables
Lasso	✓	
Debias. Lasso	✓	
Cox		✓
Gam		✓
Forest	✓	✓
Boosting	✓	✓

The final submission for the Prostate Cancer DREAM challenge from team KUStat was based on a generalized additive model using stability selected variables and the MCAR imputation scheme. Some hand tuning of the final submission was made, see our write-up ^22^ for details. The hand tuning was not implemented for this paper.

The implementation consists of a collection of supporting R functions and a main training and prediction function that fits all the 24 models on a training data set and returns the risk scores for a test data set. The assessment pipeline consists of a 5-fold cross-validation step to estimate iAUC using the training data only, and a refit step where the models are fitted to the full training dataset and risk scores are predicted for the validation dataset.

For the iAUC estimates reported in this paper, we replicated the 5-fold cross-validation 3 times and averaged the results to decrease the variation due to the random data splits. For the iAUC estimates based on the validation data we submitted predictions to the post-challenge leaderboard to assess the predictions on the 313 patients from the ENTHUSE-33 trial. We made two submissions for each model and averaged the results. Though we only found minor variations in the results for the two submissions, the double submission was done because several aspects of the model fitting rely on randomness. We wanted the results to be robust to this variation.

### Results

**Figure 2.**
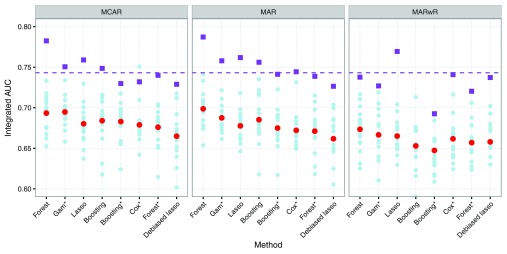
Integrated AUC for different combinations of methods evaluated by three replications of 5-fold cross-validation. Results are shown for individual folds (light blue filled circles) and averaged over all folds (red filled circles). The figure also shows iAUC on the validation data (purple filled squares) and iAUC for the reference model on the validation data (purple dashed line). The four methods marked with a * used variables chosen via stability selection, whereas the other four methods relied on implicit variable selection.

Figure 2 shows the iAUC score as estimated by cross-validation and on the validation data for all 24 combinations of methods. First, we observe that, in general, the iAUC was lower when the response was used for imputation (MARwR). The two other imputation schemes gave comparable results, and the results reported below refer to MCAR as well as MAR imputation in combination. Survival forests were overall best with iAUC around 0.78 on the validation data and just below 0.70 in the cross-validation. Debiased lasso was worst with an iAUC around 0.73 on the validation data and 0.66 in the cross-validation. The differences are small, and we also note a large variation between folds in the cross-validation indicating heterogeneity in the training data.

The generalized additive model was the best among those that relied on stability selection with iAUC around 0.76 on the validation data and 0.69 in the cross-validation. The pure lasso prediction did surprisingly well on the validation data, compared to the cross-validation results and irrespectively of the imputation method, and the computationally much more expensive boosting method was only just on par with lasso overall.

**Figure 3.**
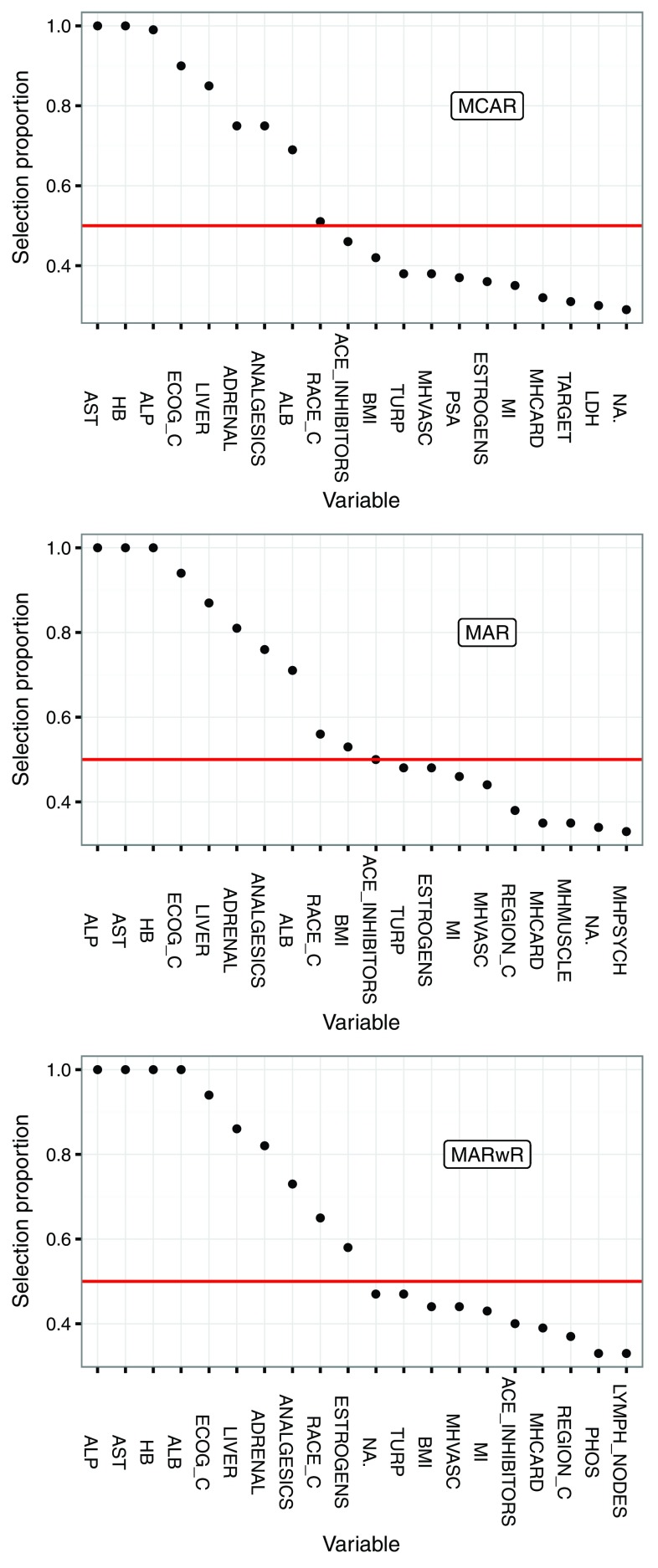
Selection proportions for the 20 most stably selected variables stratified by imputation method. The threshold of 50% (red line) was used for the final variable selection.

The results from stability selection are interesting in themselves. Figure 3 shows selection proportions for the 20 most often selected predictors for each of the imputation methods. These results are from one run of the algorithm with 100 subsamples. The variability due to the random subsampling was found to be small, though some variables would cross the (somewhat arbitrary) threshold of 50% in some runs and not in others. Figure 3 is from one of the two replications used for the validation.

The eight variables AST, HB, ALP, ECOG_C, ADRENAL, LIVER, ANALGESICS and ALB were selected in a large proportion of the subsamples, irrespective of the imputation scheme. The variable RACE_C just reached the 50% threshold for all three imputation schemes, while BMI, ACE_INHIBITORS and ESTROGENS reached the 50% threshold for some, but not all, imputation schemes. Notably, PSA was not stably selected. It is also noteworthy that ALB (which has 493 missing values) increased its selection proportion considerably when imputed using the response.

## Discussion

It is difficult to correctly evaluate how well a prognostic model will generalize. We believe that competitions like the Prostate Cancer DREAM Challenge have a positive effect on the development of systematic approaches to model evaluation. However, the big differences between the cross-validated estimates of iAUC and those obtained on the validation data demonstrate how difficult it is to generalize from one dataset to another. Such differences in predictive strength, as measured by iAUC, can be explained by differences in either the composition of the patients, or in how their survival is related to the predictors, but we cannot offer a more detailed explanation. In addition, we interpret the large variation between the cross-validation folds as evidence of a heterogeneous training dataset. This is not surprising, given that the training data is pooled from three different trials. Moreover, we noted that the majority of patients in the dataset are white and from Europe or North America. Thus it is also difficult to tell how well a prognostic model based on the Prostate Cancer DREAM Challenge data will generalize to other populations.

On the other hand, even if the absolute values of iAUC are incomparable, the rankings of the fitted models obtained by either cross-validation or validation were roughly the same. Thus we believe that our results shed light on which methods are most useful for developing prognostic survival models and for selecting variables of prognostic value.

For variable selection we believe that the poor performance of debiased lasso is a consequence of lasso generally selecting too many variables – see Chapter 8 in 23 for an extensive treatment of variable selection with lasso – and thus without shrinkage of the corresponding coefficients, debiased lasso will overfit. Stability selection is a more stringent selection criterion, which is less prone to select false positives, see Chapter 10 in 23 and 14. The variables selected by stability selection as having prognostic value also largely agree with those found in 3 and used in the reference model. One difference is that the DREAM dataset gives nuanced information on disease sites, and we found that liver and adrenal lesions, in particular, had prognostic value. The PSA variable was, on the other hand, not selected. It was a predictor in the reference model, but not a very strong one. Based on this study we therefore recommend stability selection as a reliable method for selecting prognostic variables.

For imputation of missing values the use of the response seemed to degrade predictive performance. This contradicts the recommendations in e.g. 8, which presents a simulation study showing that imputation based on inclusion of an estimate of the cumulative hazard function and the indicator of censoring is superior to a number of other imputation schemes. The framework of 8 is, however, focused on parameter estimation and hypothesis testing using multiple imputation, where the objectives differ from those of prognostic modeling. We believe that further investigations into the effect of imputation – in particular when using the response – are needed to fully understand benefits and pitfalls, but our recommendations based on this study is to avoid using the response for imputation when building prognostic models.

Finally, the best performing model – the survival forest – is the only model considered that is not based directly on the proportional hazards assumption. Thus we may speculate that this assumption could be violated.

## Conclusions

Survival forests without explicit variable selection gave the best performance overall in the cross-validation and on the validation data. When stability selection was used for explicit variable selection, the generalized additive model gave the best performance. Imputation using the response appeared to have a negative effect on predictive performance.

The four stably selected laboratory measurements AST, HB, ALP and ALB and the ECOG performance status were selected as some of the most important prognostic factors, together with liver and adrenal lesions and prior use of analgesics.

## Data and software availability

The Challenge datasets can be accessed at: https://www.projectdatasphere.org/projectdatasphere/html/pcdc

Challenge documentation, including the detailed description of the Challenge design, overall results, scoring scripts, and the clinical trials data dictionary can be found at: https://www.synapse.org/ProstateCancerChallenge

The code and documentation underlying team KUStat's challenge submission can be found at: http://dx.doi.org/10.7303/syn4260742 ^22^

The R code and documentation underlying the methods presented in this paper can be found at: https://github.com/nielsrhansen/ProstateDream. An archived version as at the time of publication is available at: http://dx.doi.org/10.5281/zenodo.50872 ^24^

## Figures and Tables

**Table S1.** Descriptions of the 93 predictor variables included in the modeling. The variables selected by stability selection by any of the imputation methods are marked with a *.

Variable name	Type	Description	Missing in training	Missing in validation
AGEGRP2	Cat. (ord)	Age Group (3 categories)	0	0
RACE_C*	Cat.	Race (5 categories)	55	0
REGION_C	Cat.	Region of the World (5 categories)	482	0
ECOG_C*	Cat. (ord)	Baseline Patient Performance Status	1	0
NON_TARGET	Binary	Baseline Non-Target Lesion(s)	0	0
TARGET	Binary	Baseline Target Lesion(s)	0	0
BONE	Binary	Baseline Bone Lesion(s)	0	0
RECTAL	Binary	Baseline Rectal Lesion(s)	0	0
LYMPH_NODES	Binary	Baseline Lymph Node Lesion(s)	0	0
KIDNEYS	Binary	Baseline Kidney Lesion(s)	0	0
LUNGS	Binary	Baseline Lung Lesion(s)	0	0
LIVER*	Binary	Baseline Liver Lesion(s)	0	0
PLEURA	Binary	Baseline Pleura Lesion(s)	0	0
OTHER	Binary	Baseline Other Lesion(s)	0	0
PROSTATE	Binary	Baseline Prostate Lesion(s)	0	0
ADRENAL*	Binary	Baseline Adrenal Lesion(s)	0	0
BLADDER	Binary	Baseline Bladder Lesion(s)	0	0
PERITONEUM	Binary	Baseline Peritoneum Lesion(s)	0	0
COLON	Binary	Baseline Colon Lesion(s)	0	0
SOFT_TISSUE	Binary	Baseline Soft Tissue Lesion(s)	0	0
ORCHIDECTOMY	Binary	Prior Orchidectomy(includes bilateral)	0	0
PROSTATECTOMY	Binary	Prior Prostatectomy	0	0
TURP	Binary	Prior Turp	0	0
LYMPHADENECTOMY	Binary	Prior Bilateral Lymphadenectomy	0	0
SPINAL_CORD_SURGERY	Binary	Prior Spinal Cord Surgery	0	0
BILATERAL_ORCHIDECTOMY	Binary	Prior Bilateral Orchidectomy	0	0
PRIOR_RADIOTHERAPY	Binary	Prior Radiotherapy	0	0
ANALGESICS*	Binary	Prior analgesics	0	0
ANTI_ANDROGENS	Binary	Prior Anti-Androgens	0	0
GLUCOCORTICOID	Binary	Prior Glucocorticoids	0	0
GONADOTROPIN	Binary	Prior Gomadotropin	0	0
BISPHOSPHONATE	Binary	Prior Bisphosponate	0	0
CORTICOSTEROID	Binary	Prior Corticosteroid	0	0
IMIDAZOLE	Binary	Prior Imidazole	0	0
ACE_INHIBITORS	Binary	Prior ACE Inhibitors	0	0
BETA_BLOCKING	Binary	Prior Beta Blocking Agents	0	0
HMG_COA_REDUCT	Binary	Prior HMG COA Reductase Inhibitors	0	0
ESTROGENS	Binary	Prior Estrogens	0	0
ANTI_ESTROGENS	Binary	Prior Anti-Estrogens	0	0
CEREBACC	Binary	Cerebrovascular accident	0	0
CHF	Binary	Congestive heart failure	0	0
DVT	Binary	Deep venous thrombosis	0	0
DIAB	Binary	Diabetes	0	0
GASTREFL	Binary	Gastroesophageal reflux disease	0	0
GIBLEED	Binary	Gastrointestinal bleed	0	0
MI	Binary	Myocardial infarction	0	0
PUD	Binary	Peptic ulcer disease	0	0
PULMEMB	Binary	Pulmonary embolism	0	0
PATHFRAC	Binary	Pathological bone fractures	0	0
SPINCOMP	Binary	Spinal cord compression	0	0
COPD	Binary	Chronic obstructive pulmonary disease	0	0
MHBLOOD	Binary	Blood & lymphatic system	0	0
MHCARD	Binary	Cardiac disorders	0	0
MHCONGEN	Binary	Congenital, familial & genetic	0	0
MHEAR	Binary	ear & Labyrinth	0	0
MHENDO	Binary	Endocrine disorders	0	0
MHEYE	Binary	Eye disorders	0	0
MHGASTRO	Binary	Gastrointestinal disorders	0	0
MHGEN	Binary	Gen. disord & admin site	0	0
MHHEPATO	Binary	Hepatobiliary disorders	0	0
MHIMMUNE	Binary	Immune system disorders	0	0
MHINFECT	Binary	Infections & infestations	0	0
MHINJURY	Binary	Injury, poison & procedural	0	0
MHINVEST	Binary	Investigations	0	0
MHMETAB	Binary	Metabolism & nutrition	0	0
MHMUSCLE	Binary	Musc./skeletal & connect tissue	0	0
MHNERV	Binary	Nervous system disorders	0	0
MHPSYCH	Binary	Psychiatric disorders	0	0
MHRENAL	Binary	Renal & urinary disorders	0	0
MHRESP	Binary	Resp., thoracic & mediastinal	0	0
MHSKIN	Binary	Skin & subcutaneous tissue	0	0
MHSOCIAL	Binary	Social circumstances	0	0
MHSURG	Binary	Surgical & medical procedures	0	0
MHVASC	Binary	Vascular disorders	0	0
BMI*	Numerical	Baseline body mass index (kg/m2)	10	1
ALP*	Numerical	Alkaline phosphatase u/l	5	2
ALT	Numerical	Alanine transaminase u/l	5	3
AST*	Numerical	Aspartate aminotransferase u/l	13	3
CA	Numerical	Calcium mmol/l	11	2
CREAT	Numerical	Creatinine umol/l	3	2
HB*	Numerical	Hemoglobin g/dl	13	4
LDH	Numerical	Lactate dehydrogenase u/l	610	4
NEU	Numerical	Neutrophils 10 ^9^/l	21	4
PLT	Numerical	Platelet count 10 ^9^/l	17	10
PSA	Numerical	Prostate specific antigen ng/ml	11	10
TBILI	Numerical	Total bilirubin umol/l	23	2
TESTO	Numerical	Testosterone nmol/l	855	2
WBC	Numerical	White blood cells 10 ^9^/l	13	4
NA.	Numerical	Sodium mmol/l	481	2
MG	Numerical	Magnesium mmol/l	510	2
PHOS	Numerical	Phosphorus mmol/l	504	2
ALB*	Numerical	Albumin g/l	493	2
TPRO	Numerical	Total protein g/l	504	2
