# Supplementary material for: Survival prognosis and variable selection: A case study for metastatic castrate resistant prostate cancer patients
Source: F1000Res. 2016 Nov 16;5:2680. [Version 1] doi: 10.12688/f1000research.8427.1 (PMC5200946; doi:10.12688/f1000research.8427.1)
Supplement: Peer review history

# Peer review: Referee response for version 1

**DOI:** 10.5256/f1000research.9071.r17680

**Published:** 2016-12-22

**Authors:** Jacob von Bornemann Hjelmborg

**Affiliations:** 1 Epidemiology, Biostatistics and Biodemography Unit, University of Southern Denmark, Odense, Denmark

This paper deals with the very hard and important problem of cancer survival prognosis with focus on evaluation of methods and data usages. Common prognosis models are assessed in a full-scale analysis, that is, in combination with imputation techniques and variable selection methods.

 The data stems from the prostate cancer DREAM challenge and the stage is set for achieving best prognosis performance in terms of the integrated AUC in case of cross-validation using the training data or for the validation dataset. The authors provide a thoroughly description of predictors and their mutual relationship.

 Combined Methods: The missingness taxonomy, which sets the range for imputation, is chosen sufficiently exhaustive and is then combined with the modelling and variable selection approaches. The chosen stability selection is appropriate and interesting given the wide range of chosen models. Some of the models have implicit variable selection and comparisons are made.

 Interestingly the debiased lasso is shown to perform rather poorly in the comparison scenario as well as the boosting methods that optimizes a concordance measure, but are low on the iAUCs, somewhat unexpected.  Explaining why the methods differ in performance is not straightforward but the general purpose of comparison is fully met and very interesting claims are provided useful for developing prognostic survival models. The stability selection procedure is recommended as a reliable method for selecting prognostic variables.

 The authors point at ad-hoc choices and "hand tuning" during the process, but manage to convey and address very well the great many choices to be made for survival prognosis in action.

 There is a comprehensive explanation of study design, chosen methods, analysis, and results. The paper is clearly written and may in combination with the supplied material serve as a very good tutorial on the topic - the case study presented seems to have very general scope for application. Papers of this type are welcomed in general.

 Misc:

 A typo of AUC in abstract.

 A specification of how mutual dependence of binary predictors is obtained.

 Not important: the estimation of the iAUC was not to be found in the supplied material - maybe a closer look is needed.

I have read this submission. I believe that I have an appropriate level of expertise to confirm that it is of an acceptable scientific standard.

# Peer review: Referee response for version 1

**DOI:** 10.5256/f1000research.9071.r17681

**Published:** 2016-11-30

**Authors:** Sarah Lemler

**Affiliations:** 1 Laboratory of Mathematics in Interaction with Computer Science, Ecole Centrale Supélec, Châtenay-Malabry, France

The authors have participated to the Prostate Cancer DREAM challenge. They report in this paper the methodology developed to predict the survival of patients with prostate cancer. They detail different methods, combining imputation techniques to replace missing values in the dataset with selection and model fitting procedures to predict the survival. They compare these methods to each other using the integrated AUC and give their conclusion about the best method in terms of prediction.  

 My opinion on the paper is the following. First of all, the paper is very well written and pleasant to read. It is clear and understandable. The procedures and methodologies followed by the authors are well explained in a concise way. In addition, the different methods considered in the paper to deal with the Prostate Cancer data are interesting not only for the dataset of the challenge, but also for other types of data. The paper gives some methodology that can be applied to any other dataset to study the survival. Lastly, the comparison of those methods shows that the best procedure is not always the one that we thought, but depends on the objective. This paper shows for example that the random survival forest is better in prediction than the Lasso, which is good in selection. As a conclusion, I strongly approve the publication of this paper.

 Some questions and little comments to improve the form:

1. Page 3, line 3: the verb "found" has to be removed.
2. Does the choice of the training, test and validation data influence the results? Would it be interesting to compare the results by changing these different samples?
3. Table 2: maybe, it would be easier for the reader to detect the differences of the age distribution in the four trials with the proportion by age class instead of the number of patients.
4. Figure 3 and paragraph 2: should we not remove the variables with lots of missing values when we do stability selection? How could you explain that the variable ALB with many missing values has been selected for example and does one really need to select this variable? 

I have read this submission. I believe that I have an appropriate level of expertise to confirm that it is of an acceptable scientific standard.
